# Interleukin-1beta (IL-1β)-induced Notch ligand Jagged1 suppresses mitogenic action of IL-1β on human dystrophic myogenic cells

**DOI:** 10.1371/journal.pone.0188821

**Published:** 2017-12-01

**Authors:** Yuki Nagata, Tohru Kiyono, Kikuo Okamura, Yu-ichi Goto, Masafumi Matsuo, Madoka Ikemoto-Uezumi, Naohiro Hashimoto

**Affiliations:** 1 Department of Regenerative Medicine, National Center for Geriatrics and Gerontology, Morioka, Oobu, Aichi, Japan; 2 Division of Carcinogenesis and Cancer Prevention, National Cancer Center Research Institute, Tsukiji, Chuo-ku, Tokyo, Japan; 3 Department of Urology, National Center for Geriatrics and Gerontology, Morioka, Oobu, Aichi, Japan; 4 Department of Mental Retardation and Birth Defect Research, National Institute of Neuroscience, Nervous, and Muscular Disorders, National Center of Neurology and Psychiatry,Ogawahigashi, Kodaira, Tokyo, Japan; 5 Department of Medical Rehabilitation, Faculty of Rehabilitation, Kobegakuin University, Ikawadani-cho, Nishi-ku, Kobe Japan; University of Minnesota Medical Center, UNITED STATES

## Abstract

Duchenne muscular dystrophy (DMD) is a severe X-linked recessive muscle disorder caused by mutations in the dystrophin gene. Nonetheless, secondary processes involving perturbation of muscle regeneration probably exacerbate disease progression, resulting in the fatal loss of muscle in DMD patients. A dysfunction of undifferentiated myogenic cells is the most likely cause for the reduction of regenerative capacity of muscle. To clarify molecular mechanisms in perturbation of the regenerative capacity of DMD muscle, we have established several NCAM (CD56)-positive immortalized human dystrophic and non-dystrophic myogenic cell lines from DMD and healthy muscles. A pro-inflammatory cytokine, IL-1β, promoted cell cycle progression of non-dystrophic myogenic cells but not DMD myogenic cells. In contrast, IL-1β upregulated the Notch ligand Jagged1 gene in DMD myogenic cells but not in non-dystrophic myogenic cells. Knockdown of Jagged1 in DMD myogenic cells restored the IL-1β-promoted cell cycle progression. Conversely, enforced expression of Jagged1-blocked IL-1β promoted proliferation of non-dystrophic myogenic cells. In addition, IL-1β prevented myogenic differentiation of DMD myogenic cells depending on Jagged1 but not of non-dystrophic myogenic cells. These results demonstrate that Jagged1 induced by IL-1β in DMD myogenic cells modified the action of IL-1β and reduced the ability to proliferate and differentiate. IL-1β induced Jagged1 gene expression may be a feedback response to excess stimulation with this cytokine because high IL-1β (200–1000 pg/ml) induced Jagged1 gene expression even in non-dystrophic myogenic cells. DMD myogenic cells are likely to acquire the susceptibility of the Jagged1 gene to IL-1β under the microcircumstances in DMD muscles. The present results suggest that Jagged1 induced by IL-1β plays a crucial role in the loss of muscle regeneration capacity of DMD muscles. The IL-1β/Jagged1 pathway may be a new therapeutic target to ameliorate exacerbation of muscular dystrophy in a dystrophin-independent manner.

## Introduction

Duchenne muscular dystrophy (DMD) is a severe X-linked recessive muscle disorder affecting 1 in 3500 boys [1]. DMD children show progressive muscle wasting and lose the ability to walk before the age of 12. DMD is caused by mutations in the dystrophin gene that is expressed in terminally differentiated myofibers. The vast majority of DMD mutations result in the complete absence of dystrophin, which damages the myofiber membrane. Then the necrosis and degeneration of myofibers is followed by massive infiltration of immune cells, chronic inflammation, and vast muscle degeneration.

Although dystrophin deficiency is the proximate cause of DMD, secondary mechanisms involving persistent inflammation and impaired regeneration may exacerbate disease progression. However, many experimental models have failed to connect the primary dystrophin mutation and secondary events. The microenvironment of dystrophic muscles includes increased numbers of immune cells that are capable of releasing numerous soluble factors including proinflammatory cytokines. Genome-wide gene expression profiling of skeletal muscle from DMD patients and mdx mice has revealed a molecular signature of dystrophinopathy, suggesting that secondary mechanisms, especially the inflammatory response, contribute to the pathogenesis [2–4]. Therefore, the inflammatory response to myofiber damage is a candidate mechanism for exacerbation of the disease [5, 6].

In addition, the inflammatory response probably affects the functions of undifferentiated myogenic cells, resulting in perturbation of muscle regeneration capacity. Actually, previous studies suggest that the proinflammatory cytokines interleukin (IL)-1, and tumor necrotic factor (TNF)-α affect both growth and differentiation of muscle satellite cells and their descendant progenitor cells. IL-1β and TNF-α activate the NF-κB signaling pathway. However, the role of IL-1β and TNF-α in myogenesis is still controversial because previous reports conflict as to whether NF-κB induces or inhibits myogenic differentiation [7–16]. These controversial results are possibly due to modification of the NF-κB signaling pathway through multiple mechanisms and crosstalk with other pathways in a cell context-dependent manner [6, 8, 17, 18].

The regenerative capacity of skeletal muscle relies largely on the presence of muscle stem cells, called muscle satellite cells [19]. Perturbation of the proliferation and differentiation processes of myogenic cells may impair the regenerative capacity of dystrophic muscles. Actually, the regenerative capacity of muscle is lost in DMD patients, presumably because muscle satellite cells undergo more frequent cell divisions and are exhausted by ongoing degeneration and regeneration cycles [1]. The capacity for proliferation and/or differentiation of myogenic cells is assumed to decline in DMD patients [20]. This loss of functions in undifferentiated myogenic cells is likely to be involved in the secondary process that exacerbates disease progression. However, it remains to be determined how and why the ability of dystrophic myogenic cells to proliferate and differentiate declines.

The mdx mouse lacks the normal dystrophin gene and is the most commonly used DMD model animal. The mdx mouse shows some features of DMD, but its life span is not grossly reduced because a vigorous regenerative response prevents the fatal loss of muscle in mdx mice. The phenotype of mdx mice suggests that mouse myogenic cells retain the ability to proliferate and differentiate in dystrophin-deficient muscles. Therefore, the mdx mouse myogenic cell is a not a very useful model of human dystrophic myogenic cells [1, 21].

Recently, dystrophin-deficient dogs have been regarded as a better model of humans with DMD because the dogs show a similar clinical course to that of DMD patients [21]. However, the regenerative capacity of canine dystrophic muscle has not been reported yet. In addition, the characteristics of canine myogenic cells remain largely unknown, and none of the established cell lines has been available. Therefore, primary cultured human myogenic cells derived from DMD patients are expected to be a breakthrough tool to elucidate the mechanisms underlying the attenuation of regenerative potential of DMD muscles. Previous studies have described the features of human dystrophic myogenic cells. Unfortunately, the reproducibility of experiments is not assured, probably because of laboratory-to-laboratory variability in the purity of populations of primary human myogenic cells. Another problem is that primary cultured human myogenic cells lose the ability to proliferate and differentiate rapidly during ongoing culture and analyses [22, 23]. We postulated that human dystrophic myogenic cells retain the molecular signature that they have in the microenvironment of DMD muscles. However, what happens in human dystrophic myogenic cells remains to be resolved.

Previously, we developed an improved immortalization protocol for human myogenic cells derived from healthy and diseased muscles [22, 23]. In the present study, to reveal the molecular mechanisms of the loss of regenerative capacity of DMD muscles, we established several human dystrophic myogenic cells from DMD patients. We found that IL-1β induced expression of a Notch ligand, Jagged1, in dystrophic myogenic cells but not in non-dystrophic myogenic cells. Jagged1 blocked IL-1β-promoted cell cycle progression and terminal muscle differentiation. A previous study suggested that a single point mutation in the promoter of the Jagged1 gene rescues the phenotype of dystrophin-deficient dogs [24]. However, the mechanisms of phenotypic modification in the rescued dogs remain to be resolved. The present results will help to reveal the role of Jagged1 in modification of a dystrophic phenotype. The susceptibility of Jagged1 gene expression to IL-1β may be crucial to attenuation of the ability to proliferate and differentiate of human dystrophic myogenic cells, resulting in exacerbation of disease progression.

## Materials and methods

### Cell culture

Primary cultured human myogenic cells were isolated and immortalized as described elsewhere [23, 25, 26]. Non-dystrophic myogenic cells were obtained from normal abdominal muscle tissues at the National Center for Geriatrics and Gerontology. Dystrophic myogenic cells were obtained from the biceps brachii muscle of Duchenne muscular dystrophy patients at the National Center of Neurology and Psychiatry (Kodaira, Japan) and Kobe University (Kobe, Japan). Muscle biopsy of patients was approved by the ethical committee of the National Center for Geriatrics and Gerontology, National Center of Neurology and Psychiatry, and Kobe University, and written informed consent was obtained from each patient before biopsy. Thirteen immortalized human myogenic cells derived from non-dystrophic and dystrophic muscles were established and analyzed (S2 Table). Immortalized human myogenic cells were maintained at 37°C under 10% CO_2_ in dishes coated with type I collagen (Sumilon, Osaka, Japan) and containing primary cultured myocyte growth medium (pmGM), which comprised Dulbecco’s modified Eagle’s medium with high glucose (hDMEM, D6429; Sigma, St. Louis, MO) supplemented with 20% fetal bovine serum (FBS) and 2% Ultroser G (Biosepra, Cedex-Saint-Christophe, France) [26]. For induction of myogenic differentiation, the medium was changed to primary cultured myocyte differentiation medium (pmDM) [26], which comprised the chemically defined medium TIS [27, 28] supplemented with 2% FBS after 48 h of culture in pmGM. To explore the effects of cytokines, cells were cultured in hDMEM supplemented with 20% FBS.

### Cell proliferation assay

Approximately 300 human myogenic cells were plated in each well of a 96-well plate containing hDMEM supplemented with 20% FBS. The cells attached to the culture vessels within 24 h after plating. The next day, the medium was changed to a test medium and cultured for another 6 d. Cells were fixed in 4% paraformaldehyde (PFA), and nuclei were stained with 2,4-diamidino-2-phenylindole dihydrochloride n-hydrate (DAPI) (1 μg/ml, Sigma). Then the number of nuclei was quantified using an In Cell Analyzer 2000 (GE Healthcare, Piscataway, NJ).

### Flow cytometry

Cells were suspended in washing buffer consisting of PBS supplemented with 2.5% FBS and stained with mouse anti-phycoerythrin-conjugated human CD56/NCAM antibody (clone AF12-7H3, Miltenyi Biotec, Bergisch Gladbach, Germany, 1:10) and allophycocyanin-conjugated human alkaline phosphatase antibody (clone B4-78, R&D Systems, Inc., Minneapolis, MN, 1:10) for 30 min at 4°C. After washing, cells were analyzed and sorted by a FACS AriaII flow cytometer (BD Biosciences).

### PCR array analysis of growth factors, NF-κB, and MAPK signaling pathway

The expression of genes related to growth factors, the NF-κB signaling pathway, or the MAP kinase signaling pathway was assessed with a Growth factors PCR Array (PAHS041D), NF-κB Signaling Pathway PCR Array (PAHS-025Z), or MAP Kinase Signaling Pathway PCR Array (PAHS-061Z), respectively (96-well plate formats of the RT^2^ Profiler™ PCR Array; SABiosciences, Frederick, MD). Synthesis of cDNA and real-time PCR was performed according to the manufacturer’s protocol using the RT^2^ First Strand Kit (SABiosciences) and RT^2^ qPCR Master Mix (SABiosciences). Cycle thresholds (CT) were uploaded to SABiosciences online PCR array data analysis software allowing for the calculation of fold change/regulation (http://www.sabiosciences.com/pcr/arrayanalysis.php). Changes in transcript abundance are expressed as the log2 ratio to control mean and designated “Fold regulation”. Thus, the software transforms fold change values less than 1 (meaning that the gene is down regulated) by returning the negative inverse.

### Quantitative reverse transcription PCR (qRT-PCR)

Total RNA was extracted with Trizol (Life Technologies, Grand Island, NY) or RNeasy kit (QIAGEN, Venio, Netherlands) and used as a template for reverse transcription using an Iscript cDNA synthesis kit (BioRad, Hercules, CA) according to manufacturers’ instructions. qPCR was performed with SsoFast Probes Supermix (Biorad), and Taqman PCR reactions were performed on CFX96 (BioRad). The amounts of mRNA were normalized to the control B2M mRNA value in pmGM or POLR2a mRNA value in pmDM and hDMEM supplemented with 20% FBS alone, respectively, because IL-1β influenced B2M mRNA values. Primers and probes were listed in a supplemental table (S3 Table). We checked the efficiency of the PCRs using diluted samples by the Pfaff method according to the manufacturer’s instructions. Determinations were performed in quadruplicate.

### Immunoblot analysis

Sample preparation and immunoblot analysis were performed as previously described [27, 28]. Immune complexes were detected by colorimetry with a BCIP/NBT detection kit (Sigma). Primary antibodies included a mouse monoclonal antibody to chicken sarcomeric myosin heavy chain (MyHC) (MF20, undiluted culture supernatant) [29], and rabbit polyclonal antibodies to Notch3 (Abcam, Cambridge, UK, 1:1000), Jagged 1 (Abcam, EPR4290, 1: 1000), and α-tubulin (Abcam, 1:1000). Secondary antibodies included alkaline phosphatase-labeled antibodies to mouse or rabbit immunoglobulin G (DAKO, Carpinteria, CA). Immune complexes on the PVDF membranes (Fluoro Trans W; Pall, Port Washington, NY) were scanned with a digital scanner (GT-9700F; Epson, Osaka, Japan) and then post-processed using Adobe Photoshop.

### Immunofluorescence analysis

Cultured cells were fixed with 4% paraformaldehyde on ice for 10 min and then incubated with primary antibodies as described elsewhere [22, 23, 26]. Primary antibodies included mouse monoclonal antibodies to chick MyHC (undiluted supernatant) and rabbit troponin T (Sigma, JLT-12, 1:200). Secondary antibodies were Cy3-conjugated antibodies to mouse IgG, Alexa488-conjugated antibodies to mouse IgG, Alexa488-conjugated antibodies to rabbit IgG, Cy3-conjugated antibodies to goat IgG (Jackson ImmunoResearch Laboratory, Bar Harbor, ME). Both primary antibodies to MyHC and those to troponin T were detected by a single secondary antibody to increase the signal intensity of differentiation markers because their fluorescent signals in human myotubes are sometimes weak, perhaps due to the species-specificity of antibodies. Cell nuclei were stained with 2, 4-diamidino-2-phenylindole dihydrochloride n-hydrate (DAPI) (1.0 μg/ml, Sigma). Samples were visualized using an inverted microscope (model IX70, Olympus, Osaka, Japan) and a CCD camera (DP50, Olympus).

A small fragment of skeletal muscle was obtained from the rectus abdominis of a 71-year-old man (sample Hu19) at the National Center for Geriatrics and Gerontology. Muscle biopsy from patients was approved by the ethical committee of the National Center for Geriatrics and Gerontology, and written informed consent was obtained from each patient before operation. Samples were immediately frozen in isopentane cooled with liquid nitrogen. Frozen muscle tissue was sectioned at a thickness of 7 μm with a cryostat (Leica Microsystems, Wetzlar, Germany), and then fixed with 4% paraformaldehyde for 5 min. Specimens were incubated with mouse monoclonal antibodies to human CD56 (clone AF12-7H3, Miltenyi Biotec, 1:11), sheep antibodies to M-cadherin (R&D Systems, Minneapolis, MN, 1:100), and rat monoclonal antibodies to laminin α-2 (clone 4H8-2, Alexis Biochemicals, Lausen, Switzerland, 1:100) at 4°C overnight and then further incubated with Cy3-conjugated antibodies to mouse IgG (Jackson ImmunoResearch Laboratories, West Grove, PA), CF488A-conjugated antibodies to sheep IgG (Biotium, Hayward, CA), and Alexa 647-conjugated antibodies to rat IgG (Invitrogen, Carlsbad, CA) for 1 h. The sections were mounted in SlowFade Gold antifade reagent DAPI (Invitrogen). Samples were visualized using confocal laser scanning microscope system LSM700 (Carl Zeiss, Oberkochen, Germany).

### Viral vector construction and viral transduction

For enforced expression of Jagged1, lentiviral vector plasmids were constructed by recombination using the Gateway system (Invitrogen). Briefly, rat Jagged1 cDNA (generously provided by G. Weinmaster) was first subcloned into an entry vector (Life Technologies). Then the segment was recombined with CSII-TRE-RfA (generously provided by H. Miyoshi) by an LR reaction (Life Technologies) to generate CSII-CMV-rJagged1. The recombinant lentiviruses with the vesicular stomatitis virus G glycoprotein were produced as described previously [30].

For suppression of Jagged1 expression, the recombinant lentiviruses encoding scrambled short hairpin (sh) RNA (sc-108080) and shRNA for Jagged1 (sc-37202-V) were obtained from a supplier (Santa Cruz Biotech., Santa Cruz, CA).

### EdU incorporation assay

Cells were cultured in DMEM supplemented with 20% FBS and incubated with 5-ethynyl-2’-deoxyuridine (EdU, 10 μM) for flow cytometry and fluorescence imaging analysis. Then the cells were fixed with 4% paraformaldehyde, and the Click-iT reaction was carried out using Click-iT Alexa Fluor 488 Imaging Kit (Life Technologies) according to the manufacturer’s instructions. For flow cytometry analysis, stained cells were analyzed by a FACS CANTO flow cytometer (Becton Dickinson, Bedford, MA). For imaging analysis, cell nuclei were stained with DAPI, and then EdU-positive nuclei were quantified using an In Cell Analyzer 2000 (GE Healthcare, Piscataway, NJ).

### Statistical analysis

All values are reported as means and standard deviations. Determinations were performed in triplicate or quadruplicate. Two group comparisons were analyzed using Student's t-test. Group comparisons were performed using one-way ANOVA.

## Results

### Human dystrophic myogenic cells were immortalized without compromising differentiation potential

**Fig 1:**
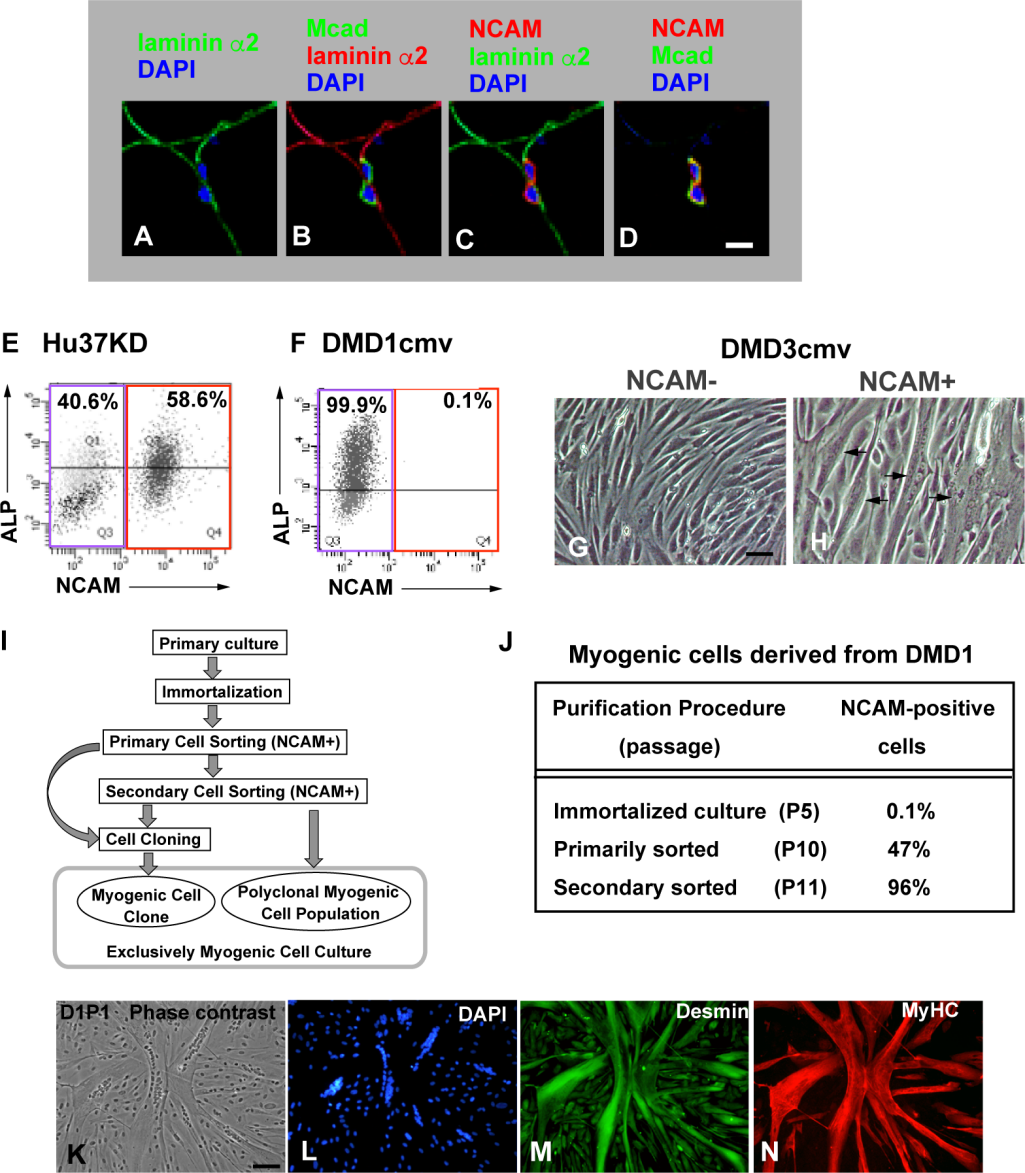
Human myogenic cells derived from non-dystrophic and dystrophic muscles were immortalized and isolated from non-myogenic cells. (A-D) Human muscle satellite cells are identified by their M-cadherin^+^/NCAM^+^ status and position under the basal lamina. DAPI, laminin α2, M-cadherin (Mcad), and NCAM were pseudocolored with different colors as indicated at the tops of the panels and then merged. Scale bar, 10 μm. (E and F) NCAM^+^ cells (red squares) were isolated from human immortalized muscle cell cultures using flow cytometry. Hu37KD is immortalized cells derived from a non-dystrophic muscle cell culture (E), whereas DMD1cmv is derived from dystrophic muscle cell culture (F). ALP, alkaline phosphatase. (G and H) Myogenic cells in the NCAM^+^ fraction were isolated. DMD3cmv is immortalized cells derived from a dystrophic muscle cell culture, and then separated into NCAM^-^ (G) and (H) cells. The cells were cultured for 7 d under the differentiation-inducing condition. Phase contrast images are shown. Arrows represent myotubes. NCAM^+^ cells were designated D3P in Fig 4 and S2 Table. Scale bar, 50 μm. (I) Procedure of isolation of human myogenic cells from primary cultured muscle cell cultures. (J) Purification and expansion of immortalized human myogenic cells. Primary cultured cells DMD1 derived from dystrophic muscle of a one-year-old boy were immortalized by the three-factor method, and 0.1% of NCAM+ cells were isolated by flow cytometry and expanded in culture. Then, 47% of NCAM+ cells in the primary sorted cell culture were isolated again and expanded. Finally, 96% of secondary sorted cells were NCAM^+^. (K-N) NCAM^+^ cells are desmin-positive myogenic cells. A single cell-derived clone D1P1 was isolated from immortalized dystrophic myogenic cell D1P derived from DMD1 cells. D1P1 expressed desmin (M) and gave rise to prominent myotubes expressing myosin heavy chain (MyHC in N) under the differentiation-inducing condition. Scale bar, 100 μm.

Primary cultured cells derived from human skeletal muscles usually contain heterogeneous cell populations. Especially, dystrophic muscle cell cultures may include many non-myogenic cells because fibroblastic cells often proliferate robustly in dystrophic muscles. Therefore, we have established experimental procedures for isolation of myogenic cells from human muscle-derived cell cultures. Human muscle satellite cells and their descendant cells express a cell surface antigen NCAM also called CD56 [31–34]. NCAM was expressed exclusively in muscle satellite cells in human skeletal muscles that were obtained from Japanese patients in the present study (Fig 1A–1D). Thus, we first tried to isolate human myogenic cells from primary cultured muscle-derived cells by flow cytometry using anti-NCAM antibodies. Primary cultured muscle-derived cells contained distinct subpopulations that were either NCAM-positive or -negative. NCAM-positive cells differentiated into myotubes under the differentiation-inducing condition, whereas NCAM-negative cells rarely gave rise to myotubes, which were probably derived from NCAM-positive cells mixed in the fraction (S1 Fig). The results demonstrated that NCAM was expressed exclusively in the muscle satellite cell lineage and that cell sorting with anti-NCAM antibodies concentrated human myogenic cells in culture. However, primary cultured human muscle cells were very fragile in the flow cytometry procedure. Thus, a significant fraction of them did not survive during the culture following cell sorting.

Our preliminary experiments showed that immortalized human myogenic cells survived the flow cytometry procedure. Thus, we first immortalized primary cultured muscle-derived cells containing both myogenic and non-myogenic cells by our three-factor method [23]. We previously found that cultured human myogenic cells express bone-liver-kidney-type alkaline phosphatase (ALP) [25]. Thus, we tried to isolate human myogenic cells from immortalized muscle-derived cells by flow cytometry using anti-NCAM and anti-ALP antibodies. However, ALP expression levels varied widely in both NCAM-positive and -negative cells (Fig 1E and 1F). Then immortalized human myogenic cells were exclusively isolated as NCAM-positive cells by flow cytometry. Immortalized human myogenic cells were also isolated from dystrophic muscle-derived cell cultures as NCAM-positive cells (Fig 1F–1H). Several dystrophic muscle-derived cultures included very small amounts of myogenic cells (Fig 1F). NCAM-negative cells were perfectly eliminated from immortalized cell cultures by two rounds of cell sorting by flow cytometry even when the primary culture contained only 0.1% of NCAM-positive cells (Fig 1I and 1J). NCAM-positive cells expressed desmin and M-cadherin and differentiated into prominent myotubes expressing the muscle differentiation marker MyHC (Fig 1K–1N; S2 Fig). The results indicate that immortalized human myogenic cells were exclusively isolated as NCAM-positive cells by flow cytometry. Our immortalization/sorting protocol provided pure populations of human myogenic cells for further analyses (Fig 1I). All the human myogenic cell lines analyzed in the present study contained myogenic cell populations alone.

### IL-1β acted as a mitogen for human non-dystrophic myogenic cells but not dystrophic myogenic cells

**Fig 2:**
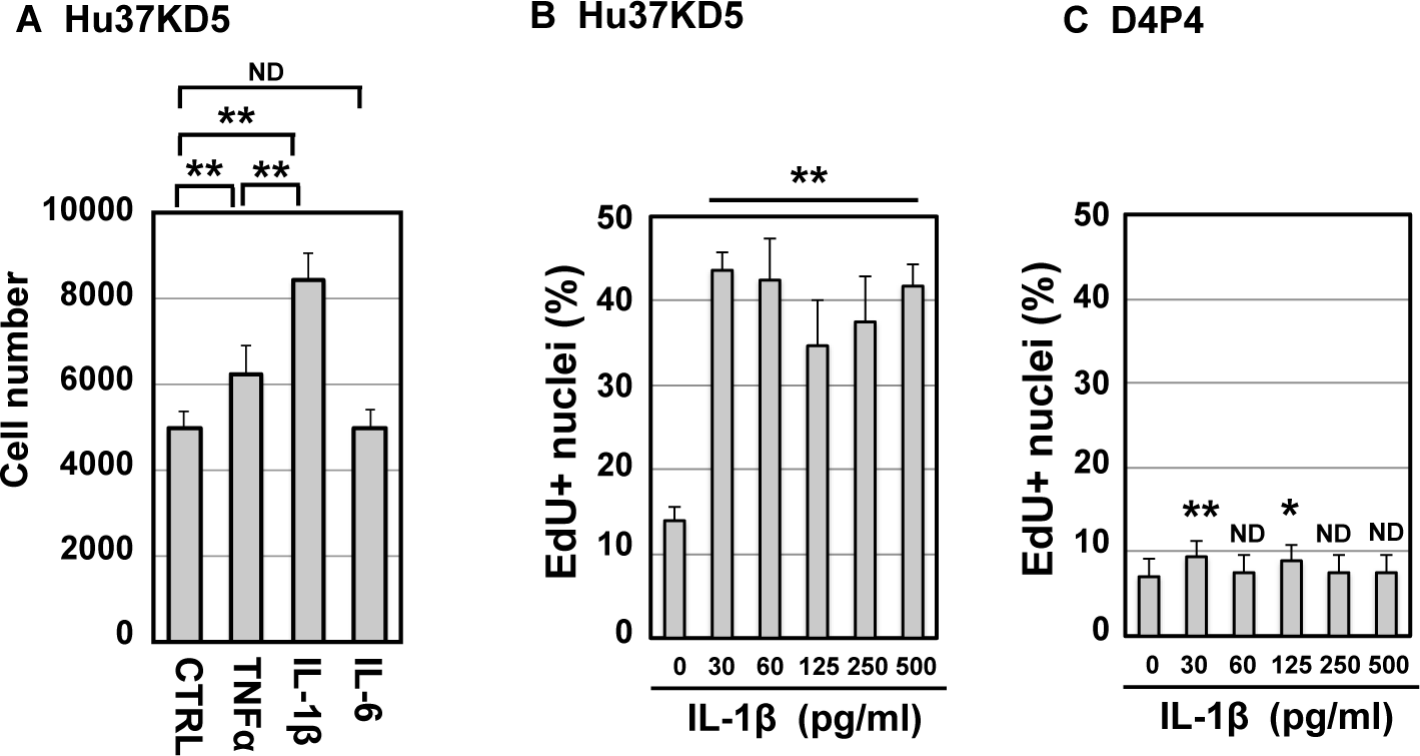
IL-1β promoted cell cycle progression of non-dystrophic myogenic cells. (A) Non-dystrophic cells (Hu37KD5) were stimulated with TNFα (500 pg/ml), IL-1β (500 pg/ml), or IL-6 (100 ng/ml) and cultured for six days. The nuclei were counted. The means and standard deviations were estimated from four independent cultures for each treatment. The statistical significance (P-value) of the difference between samples was analyzed using one-way ANOVA. ND, p>0.05; **, p<0.01. (B and C) Non-dystrophic (Hu37KD5, B) and dystrophic (D4P4, C) myogenic cell clones were treated with various concentrations of IL-1β (0–500 pg/ml) for 24 h. The cells were incubated with EdU (10 μM) for further 6 h, and the Click-iT reaction was carried out; then the cells were subjected to fluorescent image analysis. EdU-positive nuclei were counted in nine areas of each culture and the means and standard deviations were estimated. Similar results were obtained from two independent experiments, and representative data are shown. The statistical significance of the difference between the unstimulated control (0 pg/ml) and cytokine-stimulated cells were analyzed using one-way ANOVA. ND, p>0.05; *, p<0.05; **, p<0.01.

Human myogenic cells are likely to be chronically exposed to the proinflammatory cytokines TNF-α, IL-1β, and IL-6 during the repeated degeneration-regeneration cycles of DMD muscle. The inflammatory response may exacerbate the progression of muscular dystrophy. However, it remains to be determined what effects the proinflammatory cytokines have on human myogenic cells. First, we examined the effects of the proinflammatory cytokines on proliferation of human myogenic cells. Hu37KD5 cells, which were isolated from a healthy muscle, were stimulated with TNF-α, IL-1β, and IL-6, consecutively. IL-1β significantly promoted the proliferation of human myogenic cells, whereas TNF-α did slightly (Fig 2A). IL-6 did not affect the proliferation of human myogenic cells although it enhanced mouse myogenic cell growth [35]. IL-1β also acted as a mitogen in non-dystrophic myogenic cell lines other than Hu37KD5. Therefore, we focused on the action of IL-1β in further study.

To determine the role of IL-1β in growth regulation of human myogenic cells, the proliferative capacity of the non-dystrophic myogenic cell clone Hu37KD5 and a dystrophic myogenic cell clone, D4P4, was determined by incorporation of a nucleotide analogue EdU. IL-1β, even at very low concentrations, dramatically increased the number of DNA-synthesizing cells in the non-dystrophic myogenic cell culture (Fig 2B). In sharp contrast, IL-1β did not stimulate DNA synthesis in dystrophic myogenic cells (Fig 2C). In addition, even a high concentration of IL-1β (500 pg/ml) failed to stimulate the cell cycle progression of dystrophic myogenic cells. The results indicate that IL-1β acted as a mitogen for human non-dystrophic myogenic cells but not dystrophic myogenic cells.

**Fig 3:**
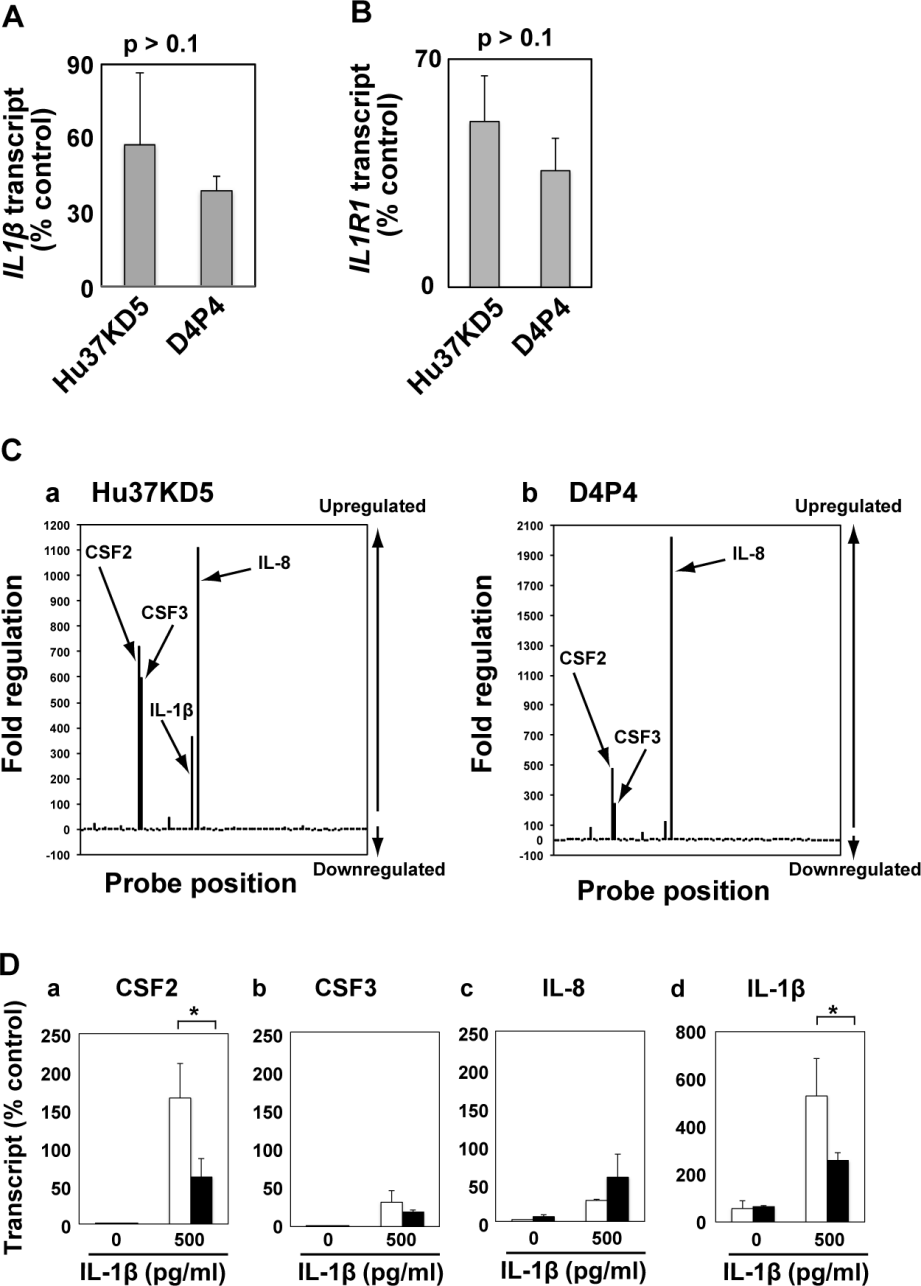
IL-1β promoted expression of NF-κB downstream genes in both human dystrophic and non-dystrophic myogenic cells. (A and B) The expressions of IL-1β (A) and IL-1 receptor (IL1R1) (B) in non-dystrophic (Hu37KD5) and dystrophic (D4P4) myogenic cell clones were analyzed by qRT-PCR. The amounts of mRNA were normalized to control POLR2a mRNA value. The means and standard deviations were estimated from four independent experiments. The statistical significance of the difference between non-dystrophic and dystrophic myogenic cells was analyzed using Student’s *t*-test. P-values were larger than 0.1 for both genes. (C) Expression profiles of members of the NF-κB signaling pathway in Hu37KD5 (a) and D4P4 (b) were determined using RT-PCR arrays after 24 h of exposure to IL-1β (500 pg/ml). Changes in transcript abundance are expressed as log2 ratio to untreated control mean and designated “Fold regulation”. (D) The expressions of CSF2, CSF3, IL-8, and IL-1β in Hu37KD5 (white column) and D4P4 (black column) were analyzed by qRT-PCR after 24 h exposure to IL-1β (500 pg/ml). The amounts of mRNA were normalized to control POLR2a mRNA values. The means and standard deviations were estimated from at least three independent experiments. Data were statistically analyzed using Student’s *t*-test. *, p<0.05.

Genes of IL-β and its receptor IL1R1 were expressed in both non-dystrophic and dystrophic myogenic cell clones when cultured in medium supplemented with FBS alone (Fig 3A and 3B). Expression levels of both genes were not significantly different in non-dystrophic and dystrophic myogenic cells although those in dystrophic myogenic cells varied somewhat. In contrast, TNF-α was not expressed in either cell.

Next, we compared gene expression profiles of NF-κB downstream members between non-dystrophic and dystrophic myogenic cells. IL-1β-induced gene expression profiles of 84 genes involved in the NF-κB signaling pathway were determined by a NF-κB Signaling Pathway PCR Array. IL-1β induced expression of 25 genes including CSF2, CSF3, IL-8, and IL-β, and decreased expression of only three genes of the NF-κB pathway in non-dystrophic myogenic cells (Fig 3Ca and S1 Table). Dystrophic myogenic cells showed similar gene expression profiles when stimulated with IL-1β (Fig 3Cb and S1 Table). Then we determined gene expression levels of CSF2, CSF3, IL-8, and IL-β in non-dystrophic and dystrophic myogenic cells. IL-1β markedly increased the expression levels of CSF2 in non-dystrophic and dystrophic myogenic cells. The expression levels of CSF2 were not likely to be critical to the mitogenic action of IL-1β on dystrophic myogenic cells, although they were significantly lower than those in non-dystrophic myogenic cells (Fig 3Da). IL-1β also increased the expression levels of CSF3 and IL-8, which were statistically similar in dystrophic and non-dystrophic myogenic cells (Fig 3Db and c). In addition, IL-1β activated its own gene expression (Fig 3Dd). The action of IL-1β was likely to be amplified by this autoactivation. Collectively, the profiling analyses of expression of the downstream target genes of NF-κB did not explain the prevention of the mitogenic action of IL-1β on human dystrophic myogenic cells.

### Jagged1 and Notch3 genes were upregulated predominantly in human dystrophic myogenic cells

IL-β had distinct biological impacts on proliferation of dystrophic and non-dystrophic human myogenic cells. Thus, we postulated another putative factor that modifies the mitogenic action of IL-1β on human dystrophic myogenic cells. To identify this putative modifier, we determined expression levels of 84 growth-related genes, including those encoding secreted factors, ligands, and receptors, in exponentially growing human non-dystrophic and dystrophic myogenic cells. We first determined the gene expression profiles in two non-dystrophic and three dystrophic immortalized myogenic cell lines derived from different patients by a Growth Factors PCR Array. We found Notch ligand Jagged1 [36] gene expression was upregulated in the three dystrophic myogenic cell lines but not in the two non-dystrophic myogenic cell lines when cultured in a growth factor–enriched medium pmGM (S3 Fig).

**Fig 4:**
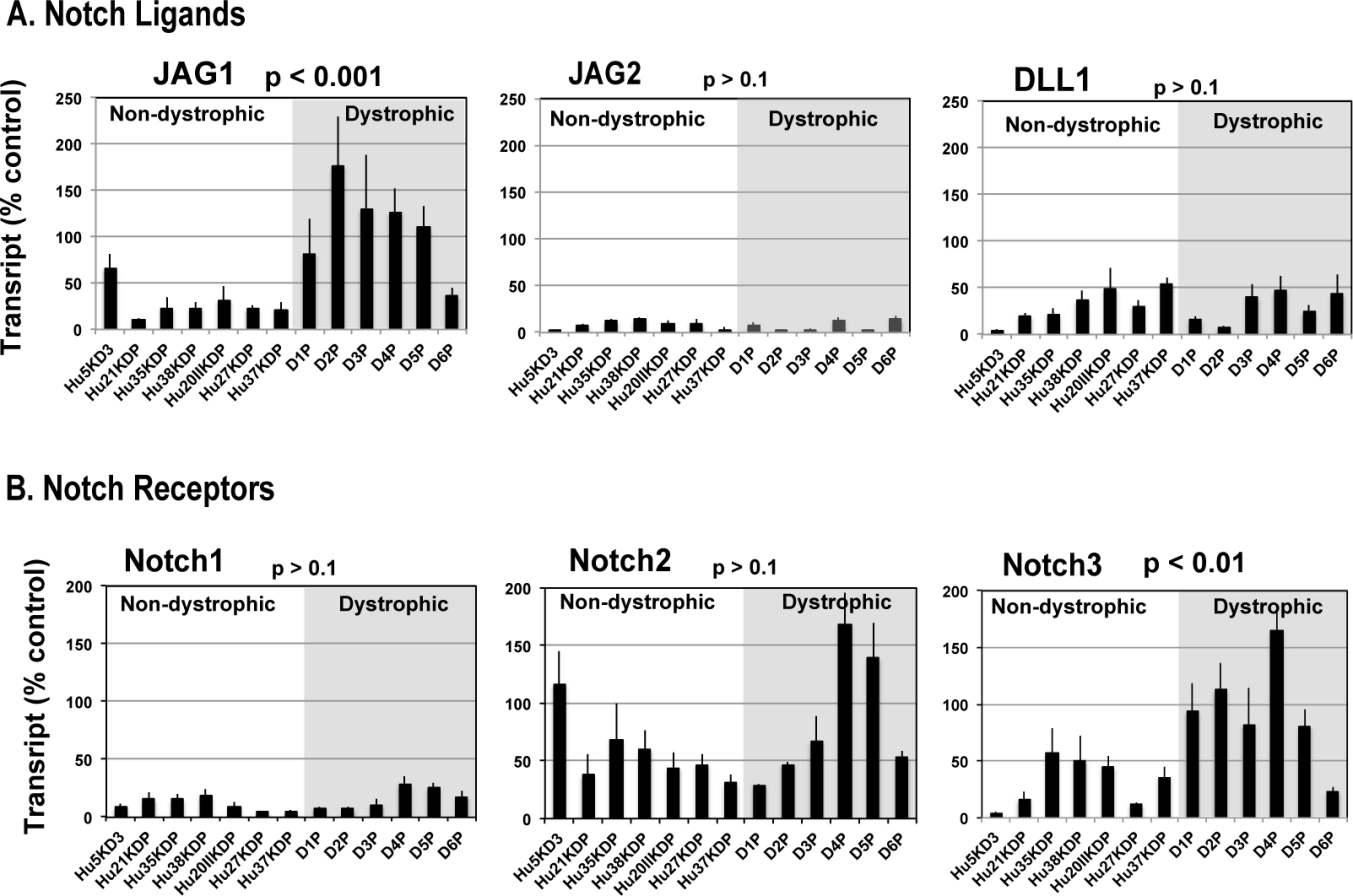
Jagged1 and Notch3 genes were upregulated in immortalized human dystrophic myogenic cells. (A and B) The expression of Notch ligands Jagged1 (JAG1), Jagged2 (JAG2), and deltaless-like1 (DLL1) (A) and Notch receptors Notch1, Notch2, and Notch3 (B) in seven non-dystrophic and six dystrophic myogenic cell lines was analyzed by qRT-PCR. Cells were cultured in growth medium pmGM. The amounts of mRNA were normalized to control B2M mRNA value. The means and standard deviations were estimated from four independent reactions for each cell line. Data were statistically analyzed using Student’s *t*-test. P values of non-dystrophic myogenic cells and dystrophic myogenic cells are shown at the tops of panels.

Then we validated gene expression levels of Notch ligands and receptors by qRT-PCR in seven non-dystrophic and six dystrophic myogenic cell lines. Jagged1 transcription was significantly upregulated in human dystrophic myogenic cells more than in non-dystrophic myogenic cells (Fig 4A). The expression levels of Jagged2 and deltaless-like1 genes remained low in both dystrophic and non-dystrophic myogenic cells (Fig 4A). Deltaless-like3 was expressed equally and deltaless-like4 was not detected in either dystrophic and non-dystrophic myogenic cells in our preliminary experiments. A Notch receptor Notch3 gene was expressed more in dystrophic myogenic cells than in non-dystrophic myogenic cells (Fig 4B). The Notch2 gene was expressed highly in two dystrophic and one non-dystrophic myogenic cell lines, but its expression levels were statistically similar in the two groups containing dystrophic and non-dystrophic myogenic cells. Notch1 transcript levels were low both in dystrophic and non-dystrophic myogenic cells. The Notch4 gene was not detected in dystrophic and non-dystrophic myogenic cells in our preliminary experiments. The results show that the gene expressions of a ligand, Jagged1, and a receptor, Notch3, in a canonical Notch signaling pathway were upregulated in human dystrophic myogenic cells. In fact, Notch3 expression is increased in skeletal muscles of DMD patients [37]. It is suggested that Jagged1 and Notch3 probably play roles in the regulation of growth and/or differentiation of human dystrophic myogenic cells although a partial contribution by Notch2 cannot be excluded.

Gene expression levels in human myogenic cells usually varied widely from person to person and from cell to cell, probably because each myogenic cell had experienced a different microenvironment in skeletal muscle. In particular, dystrophic myogenic cells may suffer severe cellular stresses depending on the condition of the patient. Actually, expression levels of Jagged1 were lower in the multiclonal dystrophic myogenic cell line D6P among six dystrophic myogenic cell lines, and higher in the non-dystrophic myogenic cell clone Hu5KD3 than in the other non-dystrophic myogenic cell lines. Despite that, dystrophic myogenic cells showed a marked tendency to express Jagged1and Notch3 under the growing condition. Thus, the microenvironment in skeletal muscle of DMD patients may modulate the expression of Jagged1and Notch3 genes in human myogenic cells.

### Low dosage of IL-1β robustly upregulated Jagged1 expression in human dystrophic myogenic cells but not in non-dystrophic myogenic cells

**Fig 5:**
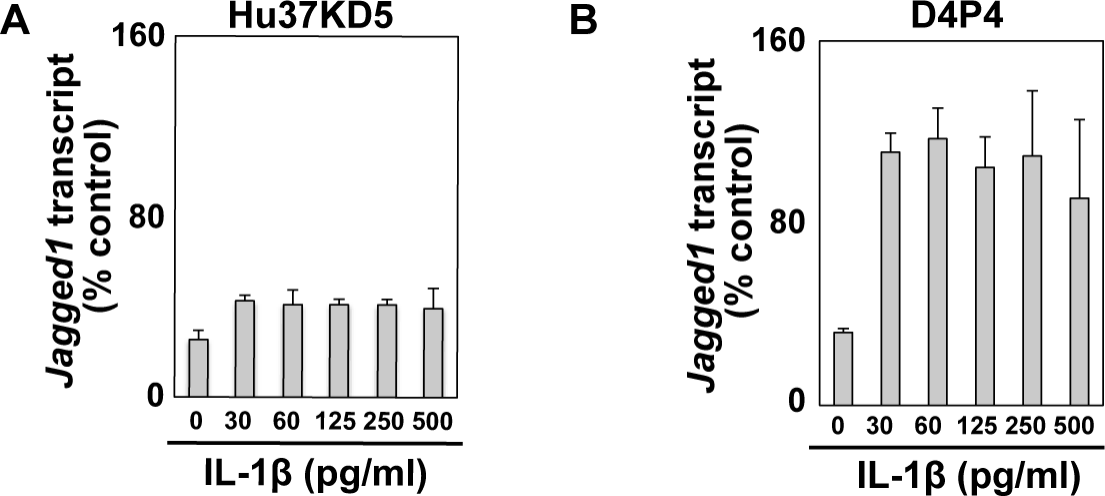
IL-1β induced expression of Jagged1 in dystrophic myogenic cells. (A and B) Non-dystrophic (Hu37KD5, A) and dystrophic (D4P4, B) myogenic cell clones were analyzed by qRT-PCR after 24 h exposure to IL-1β. The amounts of mRNA were normalized to the control POLR2a mRNA value. The means and standard deviations were estimated from at least three independent experiments. Data were statistically analyzed using one-way ANOVA. The statistical significance of the difference between unstimulated control (0 pg/ml) and cytokine-stimulated cells (30–500 pg/ml) was less than 0.01 in both cells.

We determined whether IL-1β affects Jagged1 gene expression in human myogenic cells because NF-κB triggers Jagged1 gene expression in human immune cells [38, 39]. The non-dystrophic myogenic cell clone Hu37KD5 and a dystrophic myogenic cell the clone D4P4 expressed the Jagged1 gene at similar levels when cultured in hDMEM supplemented with 20% FBS alone. When stimulated with various concentrations of IL-1β for 24 h, Jagged1 gene expression levels were markedly increased in dystrophic myogenic cells, whereas it was very slightly upregulated in non-dystrophic myogenic cells (Fig 5A and 5B). Even low concentrations (30–60 pg/ml) of IL-1β increased Jagged1 gene expression levels to high concentrations (125–500 pg/ml) in dystrophic myogenic cells. In contrast to NF-κB downstream genes (Fig 3), Jagged1 was upregulated by IL-1β exclusively in dystrophic myogenic cells.

### Jagged1 antagonized mitogenic action of IL-1β on human non-dystrophic myogenic cells

**Fig 6:**
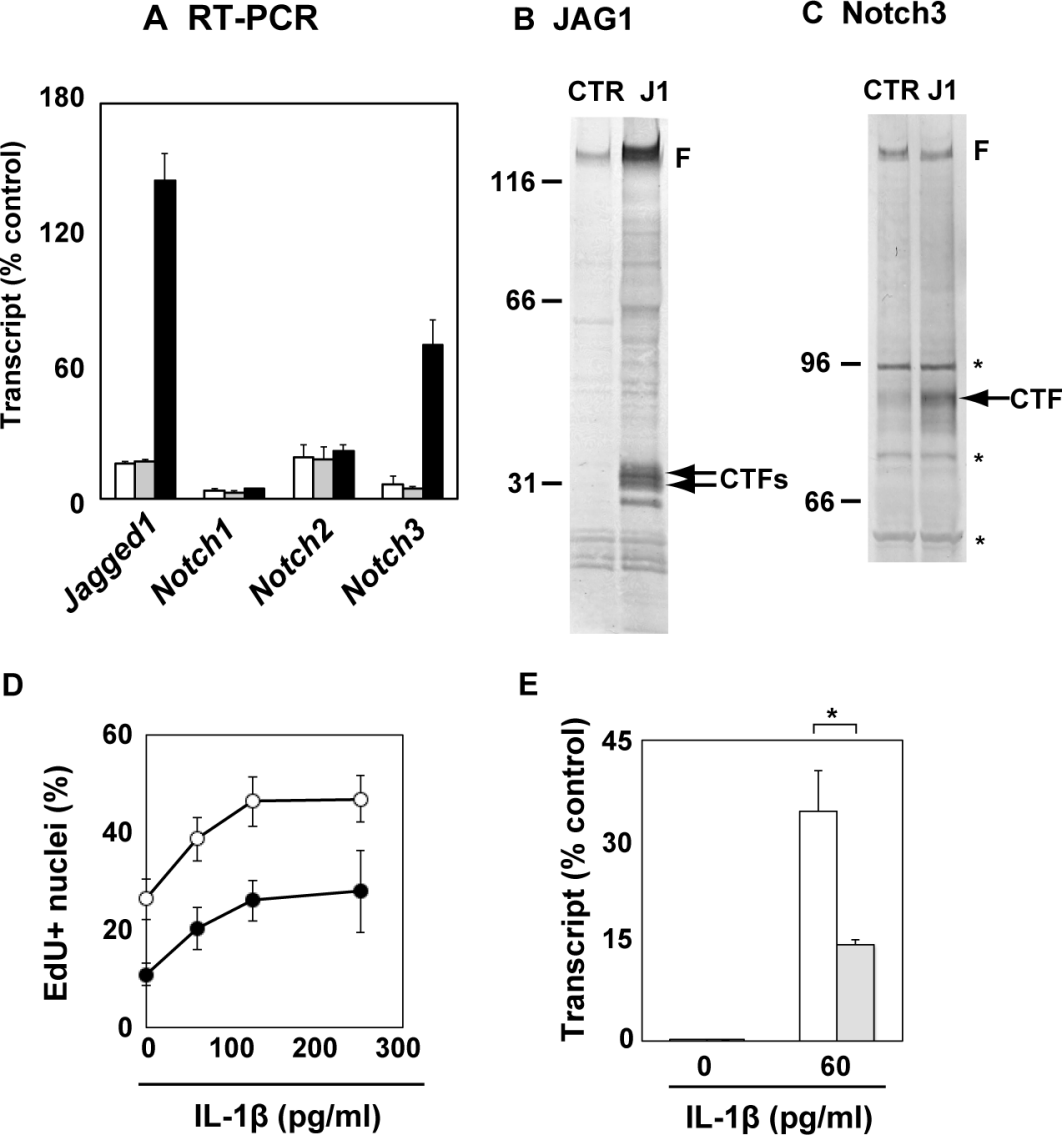
Enforced expression of jagged1 interfered with cell cycle progression and antagonized IL-1β. (A) The non-dystrophic myogenic cell clone Hu37KD5 (white column) was transduced with a control lentivirus vector (Hu37CTR, gray column) or a Jagged1-expression lentivirus (Hu37rJ1, filled column). The amounts of transcripts of Jagged1, Notch1, Notch2, and Notch3 were determined by qRT-PCR. The amounts of mRNA were normalized to control the POLR2a mRNA value. The means and standard deviations were estimated from three independent experiments. (B-C) Twenty micrograms of total proteins from Hu37CTR (CTR) and Hu37rJ1 (J1) were subjected to immunoblotting with antibodies against Jagged1 (B) and Notch3 (C). F, Unprocessed full-length protein; CTF, cytoplasmic fragment; asterisks, non-specific bands. Numbers represent the positions of protein size markers. (D) Hu37CTR (white circles) and Hu37rJ1 (filed circles) cell clones were treated with IL-1β (0–250 pg/ml) for 24 h. The cells were incubated with EdU (10 μM) for a further 6 h culture, and then subjected to fluorescence analysis. EdU-positive nuclei were counted in nine areas of each culture and the means and standard deviation were estimated. Data were statistically analyzed using one-way ANOVA. The statistical significance of the difference between the unstimulated control (0 pg/ml) and cytokine-stimulated cells (60–250 pg/ml) was less than 0.01 in both Hu37CTR and Hu37rJ1. The statistical significance of the difference between Hu37CTR and Hu37rJ1 was less than 0.01 at each dosage of IL-1β. (E) The expression of CSF2 in Hu37CTR (white column) and Hu37rJ1 (gray column) cells was analyzed by qRT-PCR after 24 h of exposure to IL-1β. The amounts of mRNA were normalized to the control POLR2a mRNA value. The means and standard deviations were estimated from three independent experiments. Data were statistically analyzed using Student’s *t*-test. *, p<0.05.

To determine whether Jagged1 interferes with the IL-1β-induced cell cycle progression, Jagged1 was forcibly expressed in non-dystrophic myogenic cells by transduction with recombinant lentiviruses encoding Jagged1. A Hu37KD5-derived clone, designated Hu37rJ1, constitutively expressed Jagged1, and the transcripts increased more than eightfold, as high as the parental Hu37KD5 (Fig 6A). Another Hu37KD5-derived clone that was transduced with a control recombinant lentivirus (empty vector), designated Hu37CTR, showed similar expression levels of the Jagged1 gene in the parental Hu37KD5. Forced expression of Jagged1 in Hu37rJ1 enhanced expression of its receptor Notch3 gene (Fig 6A). Therefore, Notch signaling might be markedly upregulated in Hu37rJ1 cells. Large amounts of Jagged1 protein were identified as both full-length and processed forms called C-terminal fragments (CTFs) (Fig 6B). A CTF of Notch3 that is an activated intracellular domain was also markedly increased in Hu37rJ1 cells (Fig 6C). The results suggest that Notch signaling was robustly activated in Hu37rJ1 cells.

Hu37CTR and Hu37rJ1 cells were stimulated with IL-1β and incubated with EdU. Then, cells synthesizing DNA were detected by fluorescence image analyses. IL-β markedly promoted cell cycle progression of Hu37CTR cells, whereas the number of DNA-synthesizing cells in Hu37rJ1 cells was much less than in Hu37CTR in the presence of lower concentrations (60–250 pg/ml) of IL-1β (Fig 6D). The number of DNA-synthesizing cells in Hu37rJ1 cells was much less than that in Hu37CTR cells even in the absence of IL-1β (represented as “0” in Fig 6D), probably because Jagged1 interferes with growth stimulating signals promoted by autocrine IL-β and/or 20% FBS. Although the low concentrations of IL-1β induced expression of CSF2, an NF-κB target gene, in Hu37CTR, the expression levels of CSF2 were significantly reduced in Hu37rJ1 cells (Fig 6E).

Taken together, it is suggested that Jagged1 antagonizes the mitogenic action of IL-1β on human myogenic cells. Promotion of Notch3 expression by Jagged1 may enhance Notch signaling. The results suggest that Jagged1 plays a key role in the modulation of IL-1β induced proliferation of human myogenic cells.

### Suppression of Jagged1 expression restored mitogenic action of IL-1β on human dystrophic myogenic cells

**Fig 7:**
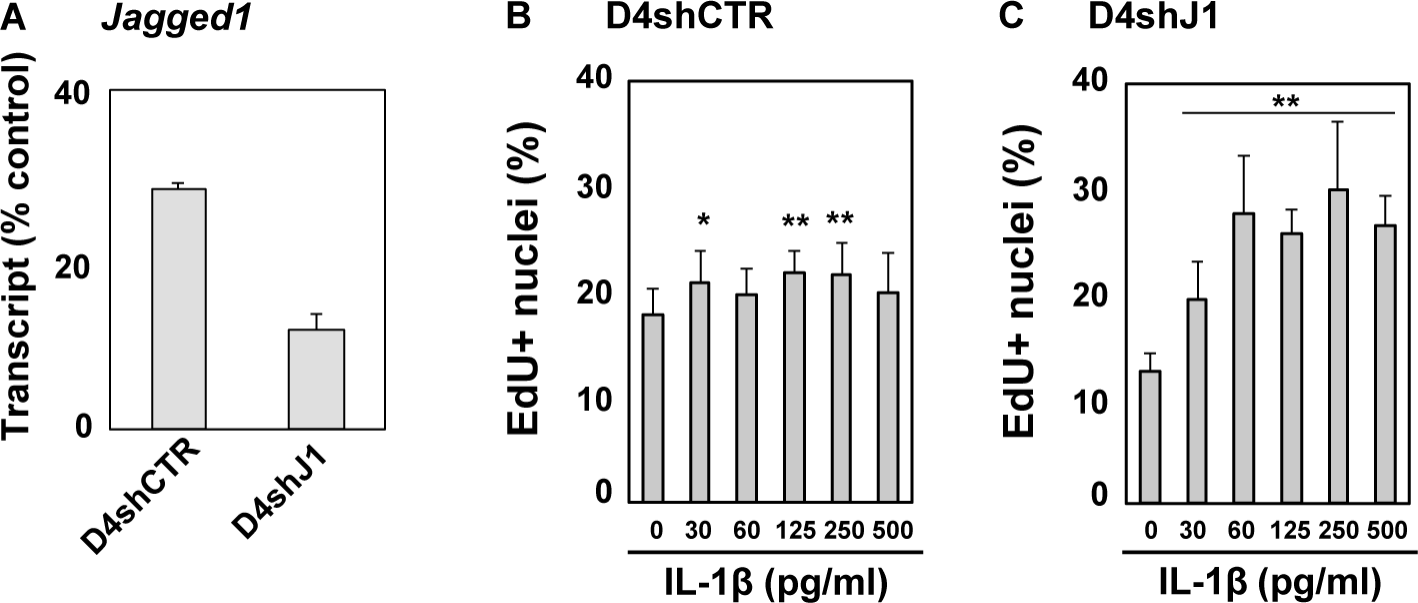
Suppression of Jagged1 expression restored mitogenic action of IL-1β in dystrophic myogenic cells. (A) The expression of Jagged1 in random shRNA (shCTR)- and shJagged1 (shJ1)-expressing D4P4-derived clones, designated D4shCTR and D4shJ1, was analyzed by qRT-PCR. The amounts of mRNA were normalized to the control POLR2a mRNA value. The means and standard deviations were estimated from three independent experiments. Data were statistically analyzed using Student’s *t*-test. The statistical significance of the difference between D4shCTR and D4shJ1cells was less than 0.01. (B and C) D4shCTR (B) and D4shJ1 (C) cells were treated with IL-1β for 24 h. The cells were incubated with EdU (10 μM) for further 6 h and then subjected to fluorescence analysis. EdU-positive nuclei were counted in nine areas of each culture and the means and standard deviations were estimated. Similar results were obtained from three independent experiments, and representative data are shown. The statistical significance of difference between the unstimulated control (0 pg/ml) and IL-1β-stimulated cells (30–500 pg/ml) was analyzed using one-way ANOVA. *, p<0.05; **, p<0.01.

To clarify whether Jagged1-induced Notch signaling is involved in quenching the IL-1β action on dystrophic myogenic cells, the expression levels of the Jagged1 gene in dystrophic myogenic cells were reduced by enforced expression of its short hairpin (sh) RNA. D4P4 cells expressing shJagged1, designated D4shJ1, exhibited reduced expression of Jagged1 (Fig 7A). D4shJ1 recaptured the ability to undergo cell cycle progression promoted by IL-1β, although a scrambled shRNA-expressing D4P4-derived clone, D4CTR, responded weakly to IL-1β (Fig 7B and 7C). In addition, the IL-1β-induced expression of the NF-κB target gene CSF2 was increased in D4shJ1 (S4 Fig). The results suggest that Jagged1 is involved in the suppression of IL-1β-induced cell cycle progression in dystrophic myogenic cells.

### IL-1β inhibited myogenesis of human dystrophic myogenic cells in a Jagged1-dependent manner

**Fig 8:**
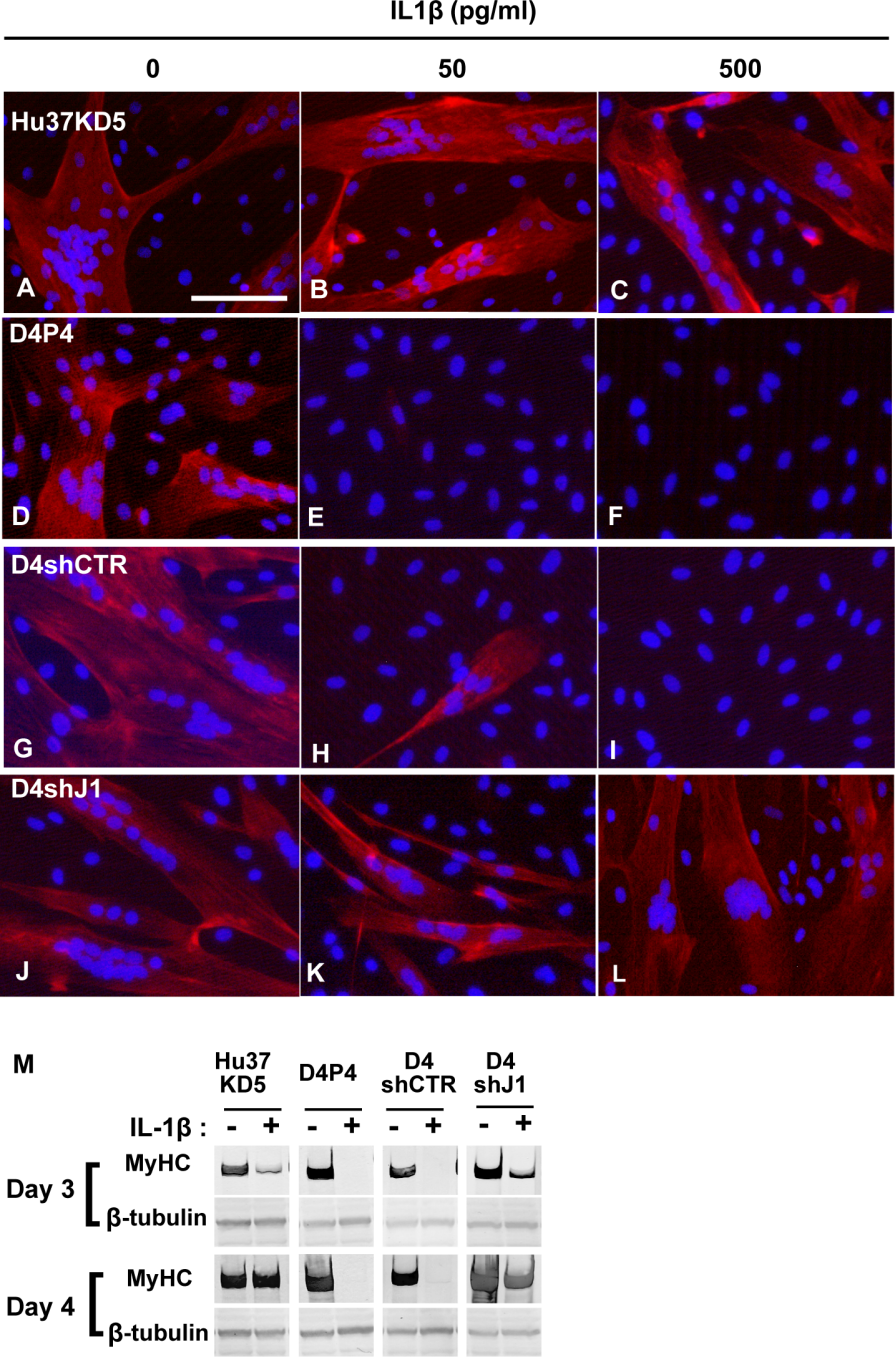
IL-1β inhibited myogenesis of dystrophic myogenic cells in a Jagged1-dependent manner. (A-R) Human non-dystrophic (Hu37KD5, A-C) and dystrophic (D4P4, D-F) myogenic cell clones and derivatives of D4P4 were stimulated with (B, C, E, F, H, I, K, and L) or without (A, D, G, and J) IL-1β (500 pg/ml) under the differentiation-inducing condition for 3 d. D4P4 cells were transduced by a lentivirus encoding random shRNA (D4shCTR, G-I) or shJagged1 (D4shJ1, J-L). Myosin heavy chain and troponin T were visualized using an inverted microscope (red). Nuclei were stained with DAPI (blue). Scale bar, 100 μm. (M) Hu37KD5, D4P4, and derivatives of D4P4 were cultured for up to 3 or 4 d under the differentiation-inducing condition with (+) or without (-) IL-1β (500 pg/ml). Twenty micrograms of total proteins was subjected to immunoblot analysis with antibodies against MyHC and β-tubulin.

It is well known that Jagged1 suppresses myogenesis (17). Activators of NF-κB also inhibit myogenesis of human and mouse myogenic cells (3–6, 8). In the next series of experiments, we determined whether the IL-1β-induced expression of Jagged1 gene is involved in the inhibition of muscle regeneration in DMD muscles. Human dystrophic and non-dystrophic myogenic cell clones were cultured in differentiation medium pmDM and stimulated with IL-1β. Non-dystrophic myogenic cells underwent myogenesis under the differentiation-inducing condition even in the presence of IL-1β, although IL-1β delayed the progression of myogenesis (Fig 8A–8C and 8M). In contrast, terminal myogenic differentiation of dystrophic myogenic cells was severely blocked exclusively when exposed to IL-1β (Fig 8D–8F and 8M). Myogenesis of dystrophic myogenic cells expressing scrambled shRNA was also blocked by IL-1β (Fig 8G–8I and 8M). However, the reduction of Jagged1 transcripts by short hairpin RNA attenuated IL-1β-dependent inhibition of myogenesis in dystrophic myogenic cells (Fig 8J–8L and 8M). The results suggest that IL-1β-induced Jagged1 prevents myogenesis and muscle regeneration in DMD muscles upon inflammation.

### Extremely high cellular stress upregulated Jagged1 in human non-dystrophic myogenic cells

The growth factor-enriched medium pmGM contains a mixture of various growth factors, cytokines, and steroids [26]. Thus, it is conceivable that the upregulation of the Jagged 1 gene in dystrophic myogenic cells depends on their hypersensitivity to extracellular signaling molecules. If that is indeed the case, signaling molecules included in pmGM might be suboptimal for non-dystrophic myogenic cells to promote Jagged1 gene expression.

We supposed that cellular stresses were possible candidates that trigger the upregulation of the Jagged1 gene in human myogenic cells because undifferentiated myogenic cells are exposed to oxidative stress and the pro-inflammatory cytokines TNF-α and IL-1β during the repeated degeneration-regeneration cycles of DMD muscle.

**Fig 9:**
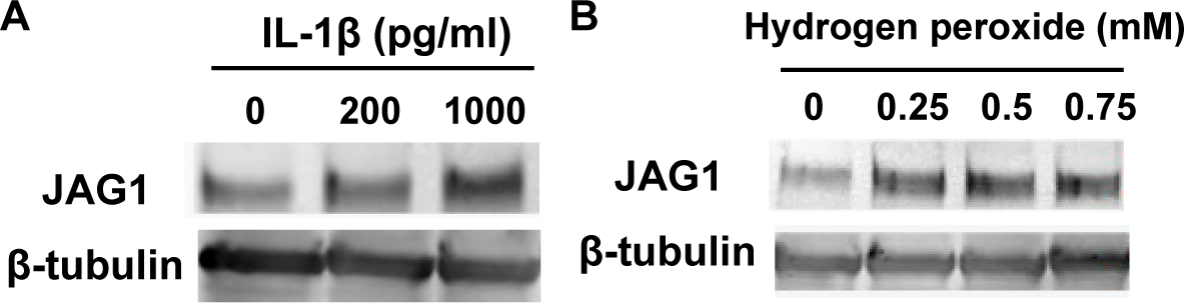
Extremely high cellular stress upregulated expression of Jagged1 in human non-dystrophic myogenic cells under growing condition. (A) Non-dystrophic myogenic cells Hu37KDP were cultured in pmGM with or without IL-1β for 24 h. (B) Hu37KDP was cultured in pmGM and exposed to hydrogen peroxide for 30 min and then further cultured in pmGM for 24 h. Twenty micrograms of total proteins was subjected to immunoblot analysis with antibodies against Jagged1. β-tubulin was used as a loading control.

An extremely high concentration of IL-1β (1 ng/ml) increased the amounts of Jagged1 protein in non-dystrophic myogenic cells (Fig 9A). Oxidative stress is another probable candidate for a Jagged1-inducing factor in human myogenic cells during chronic inflammation in DMD muscle. In fact, reactive oxygen species (ROS) generated by high concentrations of hydrogen peroxide (more than 250 μM) triggered upregulation of Jagged1 in non-dystrophic myogenic cells (Fig 9B). The results suggest that severe cellular stresses trigger upregulation of the Jagged1 gene expression in human non-dystrophic myogenic cells. Therefore, Jagged1 gene expression may be a cellular feedback response of human myogenic cells to excess stimulation with stress factors.

## Discussion

Secondary mechanisms involving impaired muscle regeneration may exacerbate disease progression in DMD patients. The present study showed that a proinflammatory cytokine, IL-1β-induced expression of a Notch ligand, Jagged1, resulted in suppression of cell cycle progression and myogenic differentiation in human dystrophic myogenic cells. Here we propose a novel molecular mechanism underlying the loss of regenerative capacity of skeletal muscles of humans with DMD.

The role of NF-κB and its activators IL-1β and TNF-α in skeletal muscle differentiation is controversial because results conflict as to whether NF-κB induces or inhibits myogenic differentiation (reviewed in [8]). Previous and the present studies suggest that NF-κB signaling is integrated with numerous other pathways [7, 15, 18]. Thus, crosstalk between NF-κB and other pathways may suggest a different interpretation of a part of the conclusions about the role of NF-κB signaling in myogenesis. The present results suggest that Jagged1 modifies the mode of action of IL-1β in human myogenic cells. In addition, previous studies suggest that TNF-α/IL-1β/NF-κB also negatively regulates myogenesis independently of the Notch signaling pathway [8], although myogenesis of human non-dystrophic myogenic cells was only temporally delayed by low concentrations of IL-1β in the present study.

Notch signaling plays a pivotal role in the regulation of growth and differentiation of myogenic cells during muscle development and regeneration [40–45]. Notch1 plays a role in the transition of muscle satellite cells from quiescence to activation. Ectopic expression of an activated Notch1 (intracellular domain of Notch1) promotes proliferation of activated satellite cells and blocks myogenic differentiation [41]. In addition, the decline of soluble Notch ligand Dll1 levels is supposed to impair the regenerative capacity of aged muscle in mice [40]. In contrast, phenotypic analysis of Notch3-deficient mice demonstrated that Notch3 suppresses proliferation of activated satellite cells during muscle regeneration, implying its antagonistic effect on Notch1 [46]. The potential role of each Notch species in the pathology of muscular dystrophy has been largely unknown. However, Notch3 is probably a main player in Notch signaling in dystrophic muscles because Notch3 but not Notch1 is exclusively upregulated in skeletal muscles of DMD patients [37]. The regenerative capacity of muscle is believed to be lost in DMD patients during repeated degeneration and regeneration cycles [1], resulting in exacerbation of progressive muscular dystrophy. The dysfunction of undifferentiated myogenic cells is a most likely cause for the reduction of regenerative capacity of muscle. Here we found upregulation of Notch3 and Jagged1 genes in undifferentiated human myogenic cells derived from DMD muscle. Jagged1 inhibited both IL-1β-triggered cell cycle progression and myogenesis probably through activation of Notch3. A knockdown of Jagged1 restored cell cycle progression and myogenesis in dystrophic myogenic cells. The present results suggest that Jagged1 reduced the ability of undifferentiated human myogenic cells to proliferate and differentiate, resulting in loss of the regenerative capacity of DMD muscle.

Our data also support a positive regulatory loop between Jagged1 and Notch3 in human myogenic cells, as also reported for ovarian cancer cells [47]. The positive regulatory loop between Jagged1 and Notch3 may markedly activate the signaling pathway downstream of Notch3, resulting in the suppression of proliferation in dystrophic myogenic cells. It should be noted that Notch3 antagonizes Notch1-promoted satellite cell proliferation [46]. Recent study suggests that Jagged1 rescues the dystrophic phenotype of dystrophin-deficient dogs and fishes [24]. Unfortunately, the mechanisms by which higher expression levels of Jagged1 protect the dogs and fishes from muscular dystrophy have remained to be resolved. Jagged1 plays distinct roles in many aspects of biology, depending on the Notch species, crosstalk with other signaling pathways, cell type/context, and animal species. The single point mutation found in the promoter of the Jagged1 gene of the rescued dogs creates a novel binding site for the myogenic regulatory factor myogenin, implying that the Jagged1 gene is possibly expressed in differentiating myoblasts and freshly differentiated myotubes/myofibers. Even if that is indeed the case, the mode of action of Jagged1 still remains to be determined. We should identify which type of cell responds to Jagged1 and which one of the Notch species is relevant for the rescue of muscular dystrophy in dystrophin-deficient dogs. In addition, it is necessary to determine whether the mutation in the Jagged1 gene affects IL-1β-induced expression of the Jagged1 gene in DMD myogenic cells. The present study demonstrated the role of IL-1β-induced Jagged1 in proliferation and differentiation of human myogenic cells. Thus, our results will contribute to understanding of the mechanisms that underlie the protective action of Jagged1 on muscular dystrophy in dogs and fishes.

**Fig 10:**
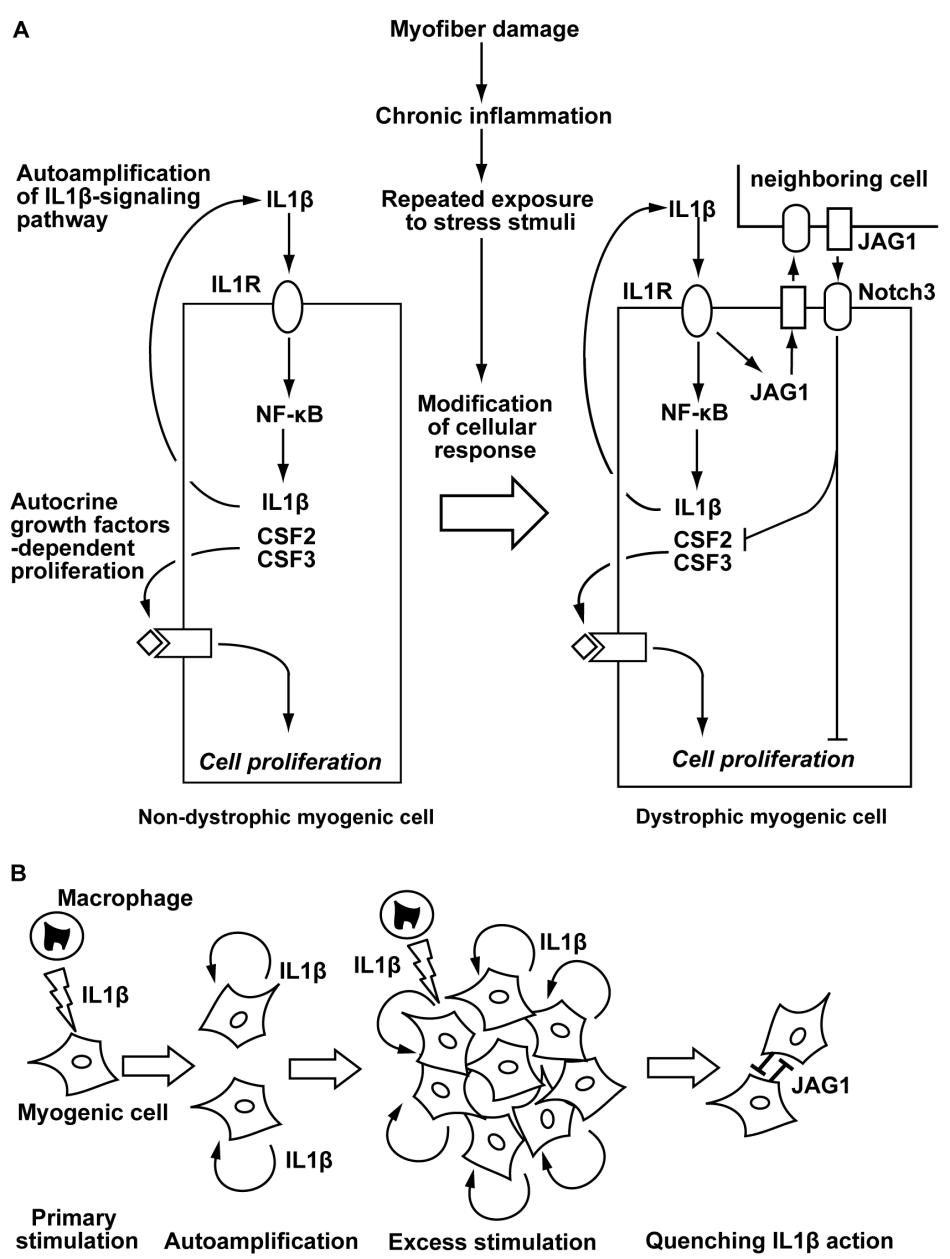
Mechanistic action of IL-1β in human myogenic cells. (A) A schematic view of the putative action of Jagged1and IL-1β in human myogenic cells. Jagged1 modulates the action of IL-1β in myogenic cells during chronic inflammation in DMD muscles. Then, the Jagged1/Notch3 signaling pathway attenuates the potential of dystrophic myogenic cells to proliferate and differentiate, whereas it is quenched in non-dystrophic myogenic cells. (B) Putative negative feedback effect of Jagged1 in dystrophic myogenic cells. Abundant/chronic exposure to IL-1β, probably derived from macrophages, induces myogenic cells to express IL-1β during repeated inflammation. The autocrine/paracrine IL-1β excessively stimulates myogenic cells, and then induces expression of Jagged1.

The susceptibility of Jagged1 to IL-1β in dystrophic myogenic cells gives us new insight into the secondary mechanisms of the pathogenesis of DMD. Myogenic cells in DMD muscles are chronically exposed to cytokines/oxidative stresses. Initially, IL-1β acts as a mitogen for human myogenic cells (Fig 10A). Then, the subsequent stimulation with IL-1β is accelerated by the autoactivation and promotes a robust increase of Jagged1 expression in primed myogenic cells, resulting in attenuation of their potential to proliferate and differentiate (Fig 10B). The susceptibility of Jagged1 in dystrophic myogenic cells is likely to be crucial to the loss of muscle regeneration capacity of DMD muscles. IL-1β-induced Jagged1 expression is likely to be a negative feedback action of the cells exposed to excess stimulation with IL-1β because higher dosages of IL-1β-increased Jagged1 levels even in human non-dystrophic myogenic cells (Fig 10B). Therefore, the susceptibility of Jagged1 in human myogenic cells is probably dependent on individual cellular histories including chronic exposure to environmental factors.

Here, we propose that Jagged1 plays a critical role in impairment of human myogenic cell functions in dystrophic muscles. The susceptibility of the Jagged1 gene to IL-1β is apparently independent of a defect of the dystrophin gene because the dystrophin gene is not expressed in undifferentiated myogenic cells. The present study suggests that the IL-1β/Jagged1 pathway will be a new therapeutic target to ameliorate exacerbation of muscular dystrophy in a dystrophin-independent manner.

## Supporting information

S1 Fig Myogenic differentiation potential of NCAM-positive cells isolated from primary culture of human muscle-derived cells. Primary cultured cells were obtained from normal abdominal muscle tissues of a 67-year-old man (Hu14) and cultured in pmGM. NCAM-positive and -negative cells were isolated from primary cultured cells at passage 7 by flow cytometry using anti-NCAM antibodies, then cultured in pmDM for 7 d. Myotubes developed exclusively in NCAM-positive cell cultures. Phase contrast images are shown. Scale bar, 100 μm.
(TIF)

S2 Fig Expression of M-cadherin in immortalized NCAM-positive cells isolated from dystrophic muscle. Primary cultured cells were obtained from DMD muscle of a 14-month-old boy (DMD3) and immortalized by the three-factor method. NCAM-positive cells were isolated from immortalized DMD3 cells (DMD3cmv) by flow cytometry, then cultured in pmDM for 7 d. NCAM-positive cells differentiated into myotubes expressing M-cadherin. Nuclei were detected by DAPI. The same field is shown.
(TIF)

S3 Fig Gene expression profiles of human dystrophic and non-dystrophic myogenic cells by a growth factors PCR array. Expression levels of 84 growth-related genes were determined in two non-dystrophic (Hu5KD3 and Hu37KDP) and three dystrophic (DMD1P, DMD2P, and DMD3P) human myogenic cell lines. Expression levels of 17 genes are shown as % of control genes.
(TIF)

S4 Fig Expression of CSF2 in Jagged1-knockdown dystrophic myogenic cells. The expressions of CSF2 in D4shCTR (white column) and D4shJ1 (gray column) were analyzed by qRT-PCR after 24 h of exposure to IL-1β (500 pg/ml). The amounts of mRNA were normalized to control the POLR2a mRNA value. Experimental conditions are the same as those in Fig 7. Statistical significance was analyzed using Student’s *t* test. *, p <0.05.
(TIF)

S1 Table Up- and downregulated genes in NF-κB pathway after stimulation with IL-1β. Fold-Change (2^(- Delta Delta Ct)) is the normalized gene expression (2^(- Delta Ct)) in the Test Sample (IL-1β-treated cultures) divided by the normalized gene expression (2^(- Delta Ct)) in the Control Sample (IL-1β-untreated cultures). Fold-change values greater than two are indicated in red; fold-change values less than 0.5 are indicated in blue.
(PDF)

S2 Table Immortalized human myogenic cell lines. (PDF)

S3 Table Primers and probes for qRT-PCR. Numbers represent probes from Universal Probe library (Roche).
(PDF)
