# Supplementary figures and images for: Interleukin-1beta (IL-1β)-induced Notch ligand Jagged1 suppresses mitogenic action of IL-1β on human dystrophic myogenic cells
Source: PLoS One. 2017 Dec 1;12(12):e0188821. doi: 10.1371/journal.pone.0188821 (PMC5711031; doi:10.1371/journal.pone.0188821)

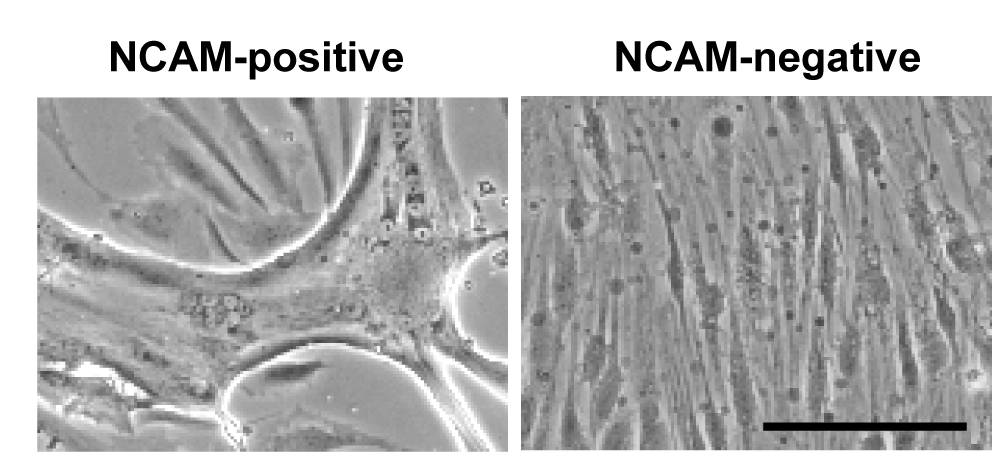

Supplement: S1 Fig — Primary cultured cells were obtained from normal abdominal muscle tissues of a 67-year-old man (Hu14) and cultured in pmGM. NCAM-positive and -negative cells were isolated from primary cultured cells at passage 7 by flow cytometry using anti-NCAM antibodies, then cultured in pmDM for 7 d. Myotubes developed exclusively in NCAM-positive cell cultures. Phase contrast images are shown. Scale bar, 100 μm. (TIF) [file pone.0188821.s001.tif]

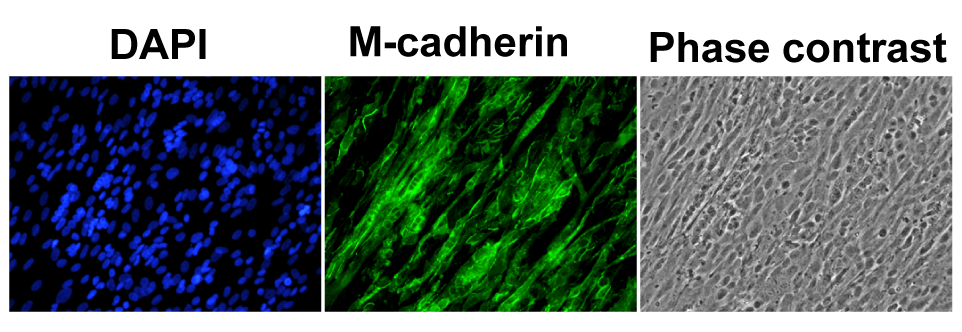

Supplement: S2 Fig — Primary cultured cells were obtained from DMD muscle of a 14-month-old boy (DMD3) and immortalized by the three-factor method. NCAM-positive cells were isolated from immortalized DMD3 cells (DMD3cmv) by flow cytometry, then cultured in pmDM for 7 d. NCAM-positive cells differentiated into myotubes expressing M-cadherin. Nuclei were detected by DAPI. The same field is shown. (TIF) [file pone.0188821.s002.tif]

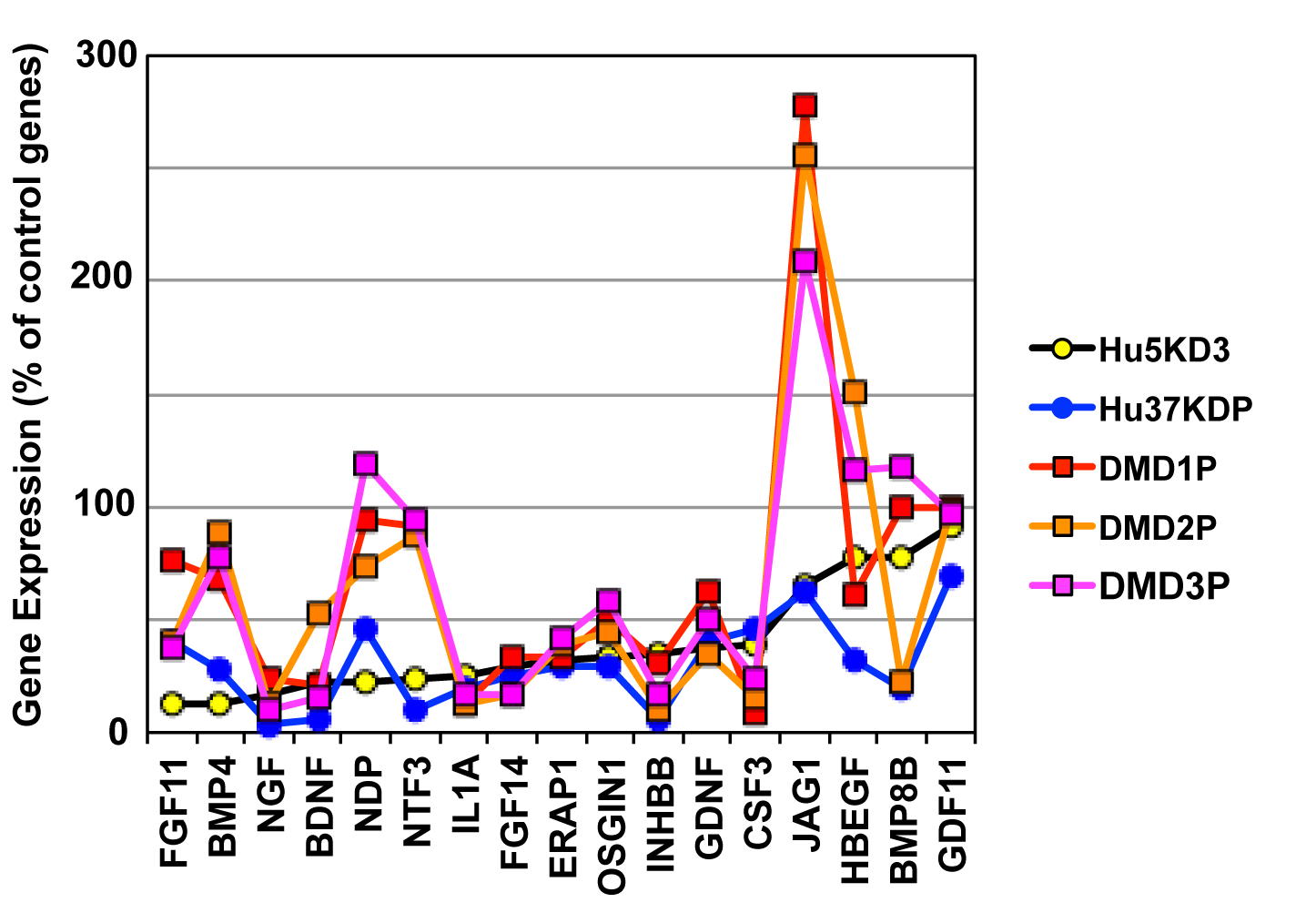

Supplement: S3 Fig — Expression levels of 84 growth-related genes were determined in two non-dystrophic (Hu5KD3 and Hu37KDP) and three dystrophic (DMD1P, DMD2P, and DMD3P) human myogenic cell lines. Expression levels of 17 genes are shown as % of control genes. (TIF) [file pone.0188821.s003.tif]

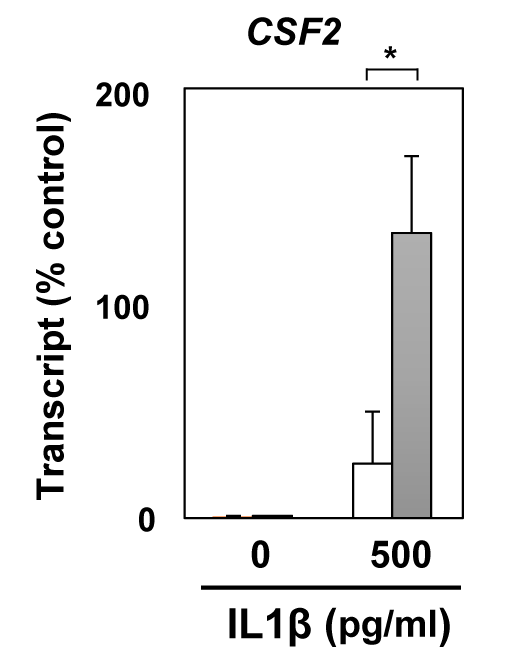

Supplement: S4 Fig — The expressions of CSF2 in D4shCTR (white column) and D4shJ1 (gray column) were analyzed by qRT-PCR after 24 h of exposure to IL-1β (500 pg/ml). The amounts of mRNA were normalized to control the POLR2a mRNA value. Experimental conditions are the same as those in Fig 7. Statistical significance was analyzed using Student’s t test. *, p <0.05. (TIF) [file pone.0188821.s004.tif]
