# Supplementary material for: Interleukin-1beta (IL-1β)-induced Notch ligand Jagged1 suppresses mitogenic action of IL-1β on human dystrophic myogenic cells
Source: PLoS One. 2017 Dec 1;12(12):e0188821. doi: 10.1371/journal.pone.0188821 (PMC5711031; doi:10.1371/journal.pone.0188821)
Supplement: S1 Table — Fold-Change (2^(- Delta Delta Ct)) is the normalized gene expression (2^(- Delta Ct)) in the Test Sample (IL-1β-treated cultures) divided by the normalized gene expression (2^(- Delta Ct)) in the Control Sample (IL-1β-untreated cultures). Fold-change values greater than two are indicated in red; fold-change values less than 0.5 are indicated in blue. (PDF) [file pone.0188821.s005.pdf]

S1 Table

NF-kB Pathway 20%FBS
Hu37KD5 IL-1B+/-

Position	Symbol	Fold Change
D04	IL8	1111.9994
B08	CSF2	719.2159
B09	CSF3	597.8781
D02	IL1B	366.0602
C06	ICAM1	45.8064
A05	BCL2A1	22.2792
B02	CCL5	15.2875
G03	TNFAIP3	11.1986
F09	TLR2	10.0745
A09	BIRC3	8.8638
D06	IRAK2	7.1358
E04	NFKBIA	6.662
D01	IL1A	5.7085
E03	NFKB2	4.6918
D07	IRF1	3.1102
E12	RELB	2.9499
E02	NFKB1	2.4775
A11	CASP1	2.434
C05	HMOX1	2.4029
E05	NFKBIB	2.193
A07	BCL3	2.1276
G02	TNF	2.1273
E10	REL	2.0896
D03	IL1R1	2.0178
G11	TRAF3	2.0119
B11	EGR1	0.4877
H01	ACTB	0.4269
C01	F2R	0.3928

D4P4 IL-1B+/-

Position	Symbol	Fold Change
D04	IL8	2023.3892
B08	CSF2	474.387
B09	CSF3	241.3453
D02	IL1B	126.3974
B01	CCL2	86.9081
C06	ICAM1	55.2903
B02	CCL5	15.6428
F10	TLR3	8.5079
G03	TNFAIP3	8.4231
A11	CASP1	8.2617
A05	BCL2A1	6.7464
F03	STAT1	4.5722
G07	TNFSF10	4.551
F09	TLR2	4.316
D07	IRF1	4.2009
A09	BIRC3	4.1855
B07	CSF1	3.4636
G02	TNF	3.3326
E03	NFKB2	3.1645
E04	NFKBIA	3.0541
D06	IRAK2	2.8767
D01	IL1A	2.4818
A07	BCL3	2.417
E12	RELB	2.066
E01	MYD88	2.0507
E02	NFKB1	2.0402
H09	RTC	0.4736
H08	RTC	0.4679
C01	F2R	0.4677
