# Supplementary material for: Interleukin-1beta (IL-1β)-induced Notch ligand Jagged1 suppresses mitogenic action of IL-1β on human dystrophic myogenic cells
Source: PLoS One. 2017 Dec 1;12(12):e0188821. doi: 10.1371/journal.pone.0188821 (PMC5711031; doi:10.1371/journal.pone.0188821)
Supplement: S2 Table — (PDF) [file pone.0188821.s006.pdf]

S2 Table. List of immortalized human myogenic cell lines.

Cell ID	Gender	Age	Comments
Hu5KD3 (KD3)	Female	42y	isolated clone (Shiomi et al., 2011)
Hu21KDP	Male	50y	polyclonal cell population
Hu35KDP	Male	54y	polyclonal cell population
Hu38KDP	Male	56y	polyclonal cell population
Hu20IIKDP	Male	75y	polyclonal cell population
Hu27KDP	Female	86y	polyclonal cell population
Hu37KDP	Female	21y	polyclonal cell population
Hu37KD5	Female	21y	isolated clone from Hu37KDP
D1P	Male	1y	polyclonal cell population
D2P	Male	5m	polyclonal cell population
D3P	Male	1y2m	polyclonal cell population
D4P	Male	4y	polyclonal cell population
D4P4	Male	4y	isolated clone from D4P
D5P	Male	1y10m	polyclonal cell population
D6P	Male	7y	polyclonal cell population
