# Supplementary material for: Interleukin-1beta (IL-1β)-induced Notch ligand Jagged1 suppresses mitogenic action of IL-1β on human dystrophic myogenic cells
Source: PLoS One. 2017 Dec 1;12(12):e0188821. doi: 10.1371/journal.pone.0188821 (PMC5711031; doi:10.1371/journal.pone.0188821)
Supplement: S3 Table — Numbers represent probes from Universal Probe library (Roche). (PDF) [file pone.0188821.s007.pdf]

S3 Table. List of primers

gene name	Primer	Amplicon (nt)	Probe: Numbers represent probes from Universal Probe Library (Roche).
POLR2a	ttgtgcaggacacactcaca<br>caggaggttcatcacttcacc	83	#1
B2M	ttctggcctggaggctatc<br>tcaggaaatttgactttccattc	86	#42
GAPDH	agccacatcgctcagacac<br>gcccaatacgaccaaatcc	66	#60
NOTCH1	cgcacaaggtgtcttccag<br>aggatcagtggcgtcgtg	87	#85
NOTCH2	ggcagactggtgacttcactt<br>ctctcacaggtgctcccttc	67	#8
NOTCH3	gccaagcggctaaaggta<br>cactgacggcaatccaca	65	#30
JAG1	gaatggcaacaaaacttgcat<br>agccttgtcggcaaatagc	71	#42
JAG2	tgggactgggacaacgatac<br>atgcgacactcgctcgat	60	#17
DLL1	gtggggagaaagtgtgcaa<br>gtcacaaaatccatgctgctc	94	#20
CSF2	tctcagaaatgtttgacctccag<br>gcccttgagcttggtgag	98	#1
IL1b	acctctgccctctggatgg<br>gaaagaaggtgctcaggtcattc	165	FAM-acgaatctccgaccaccactacagc-TAMRA
IL1R1	ctgcaaagacaaggttctggaag<br>ccttagtggctggtgacagtaac	100	FAM-atgccagtccagcgacggtcacct-TAMRA
